# *TP53* Is a Potential Target of Juglone Against Colorectal Cancer: Based on a Combination of Molecular Docking, Molecular Dynamics Simulation, and In Vitro Experiments

**DOI:** 10.3390/cimb47060439

**Published:** 2025-06-10

**Authors:** Yunting Deng, Yanan Zhang, Xinghai Chen, Weiming Wang, Jinhai Huo

**Affiliations:** Heilongjiang Academy of Traditional Chinese Medicine, Harbin 150036, China; 13060204342@163.com (Y.D.); 13827475451@163.com (Y.Z.); cxh15382098368@163.com (X.C.)

**Keywords:** Juglone, colorectal cancer, molecular docking, cell experiments, p53 protein, molecular dynamics simulations

## Abstract

Background: Colorectal cancer is the third most common cancer worldwide, accounting for about 10% of all cancer cases. There is an urgent need to improve treatment outcomes and survival rates for colorectal cancer. Juglone is an anthraquinone with anti-inflammatory, antiviral, and anti-cancer properties that have shown promise in inhibiting tumor cell growth. Objectives: This study aims to explore the mechanism behind Juglone’s anti-cancer effects on colorectal cancer. Methods: Network pharmacology, molecular docking and molecular dynamics simulation were used to explore the specific targets of Juglone in the treatment of colorectal cancer. For in vitro validation, we used the CCK–8 (Cell Counting Kit–8) method, flow cytometry, ROS (Reactive Oxygen Species) detection, and Western blot analysis to assess the survival ability of colorectal cancer cells and validate the expression of proteins most closely associated with the pathways. Results: Network pharmacology identified *TP53* as a key target of Juglone, involved in anti-tumor pathways. Molecular docking and molecular dynamics simulations showed that the p53 has strong affinity and stability with Juglone. Results from cytotoxicity experiments, flow cytometry, ROS detection, and Western blotting indicated that the anti-colorectal cancer effect of Juglone depends on concentration and is mediated by promoting intracellular ROS generation and upregulating the expression level of p53 protein, thereby inhibiting the progression of colorectal cancer. Conclusions: Juglone can achieve anti-colorectal cancer effects by increasing ROS levels and regulating the p53 protein.

## 1. Introduction

Colorectal cancer (CRC) is a common type of gastrointestinal tumor. As of 2020, the standardized incidence and mortality rates of colorectal cancer in our country are 23.9 and 12.0 per 100,000 people, respectively, ranking second in incidence and fifth in mortality among all malignant tumors [1]. Studies have shown a steady increase in CRC incidence among individuals under 50 years of age. Globally, CRC incidence has risen in both men and women, with an overall trend toward younger populations [2,3,4]. The pathogenesis of colorectal cancer involves multiple factors and steps. Changes in lifestyle, such as increased antibiotic use and obesity, may contribute to the rising incidence of early-onset CRC [5,6]. Currently, CRC treatment methods include surgery, endoscopic therapy, targeted therapy, and immunotherapy [7,8]. However, the efficacy of these treatments varies among individuals. Nonetheless, the aforementioned treatments have notable shortcomings, including increased drug resistance, severe adverse reactions, and unclear pathogenesis, which may involve complex interactions among multiple pathways. These issues have garnered significant attention [9].

Juglone is an anthraquinone molecule extracted from the mature exocarp of walnut and black walnut plants, known for its anti-tumor and antibacterial properties. Some researchers have used MTT(Methylthiazolyldiphenyl-tetrazolium bromide) assays and Western blot techniques to study the effects of Juglone on human ileocecal cancer cells, focusing on adhesion and matrix metalloproteinase activity. The results showed that Juglone inhibited the adhesion of human ileocecal cancer cells in a dose-dependent manner. It significantly reduces the extracellular matrix of these cells and is expected to inhibit their invasion and metastasis [10]. Furthermore, to explore the combined therapeutic effects of Juglone and related drugs, researchers combined Juglone with oxaliplatin in experiments with four colorectal cancer cell lines (LOVO, SW480, etc.). They found that both Juglone and oxaliplatin significantly inhibited colorectal cancer cells, with Juglone exhibiting a stronger effect. When used together, the inhibition was enhanced regardless of their ratio, and the effect was dose-dependent [11]. Based on this, Diels–Alder reactions were conducted between 3,6-dimethoxybenzoyl intermediates and furans, leading to further acid-catalyzed intramolecular rearrangements and CAN-mediated oxidation(Cerium (IV) ammonium nitrate mediated oxidation). This resulted in the synthesis of novel 5-benzylcarbachol quinones from 5-benzylcarbachol. The effect of this derivative on the proliferation of human colorectal cancer cells was observed. Results indicated that 5-benzylhomoquinone promoted apoptosis in human colorectal cells by arresting the HCT15 (human colon cancer cell line) cell cycle in the G0/G1 phase [12]. In recent studies, some researchers investigating the anti-pancreatic cancer effects of Juglone in combination with anti-thrombotic sodium found that the expression of anti-apoptotic genes decreased, and the expression of apoptotic genes increased after treatment at all doses [12]. In addition, there were researchers [13] found that in vitro and in vivo experiments further confirmed that Juglone induced inhibition of iron death in glioblastoma through activation of p38/MAPK phosphorylation and negative regulation of the Gpx4 signaling pathway. In an anti-cancer study in human ovarian cancer cells, Juglone was found to induce G2/M cell cycle arrest, and Juglone was shown to inhibit the migration and invasion of cells in a wound healing and a transwell assay, suggesting that Juglone has antimetastatic potential [14]. These studies suggest that Juglone has great potential for anti-tumor activity.

Network pharmacology, based on systems biology theory, analyzes biological system networks and emphasizes the multi-pathway regulation of signaling pathways. This approach aims to enhance drug efficacy, reduce toxic side effects, and improve the success rate of new drug clinical trials. Network pharmacology relies on extensive databases, which are used to enhance and complement biological data [15]. Reports indicate that cyberpharmacology has become a reliable method for establishing “disease gene/compound target” networks and providing intuitive maps of target prediction mechanisms. This high-throughput approach helps reveal the regulatory principles of small molecules [16]. Molecular docking predicts the optimal orientation and binding affinity of small molecules (ligands) to target proteins, assisting in the identification of promising candidates by simulating and scoring their interactions at binding sites. Molecular dynamics simulations provide a time-dependent view of these interactions, revealing properties such as stability, conformational changes, and other properties of ligand-protein complexes. Together, these technologies can improve the understanding of molecular mechanisms, guide compound optimization, and improve the efficiency and accuracy of the drug development process [17]. Previous studies have shown that Juglone has anti-tumor effects, but research on its specific anti-tumor mechanisms is limited. Therefore, this study employed state-of-the-art network pharmacology, molecular docking, and molecular dynamics simulation to predict Juglone’s potential targets in treating colorectal cancer (CRC). Additionally, CCK-8 cell cytotoxicity assay and Western blot protein expression analysis were used to explore its mechanism, aiming to provide a foundation for CRC treatment.

## 2. Materials and Methods

### 2.1. Network Pharmacological Analysis of Juglone

#### 2.1.1. Juglone and CRC Gene Collection and Target Prediction

Relevant ingredient information was identified using the TCM Systematic Pharmacology Database and Analysis Platform (TCMSP (https://www.tcmsp-e.com/load_intro.php?id=43, accessed on 4 June 2025)) and SwissTargetPrediction (http://swisstargetprediction.ch/, accessed on 4 June 2025). CRC-related targets were searched using the keyword “colorectal cancer”. Disease-related genes for CRC were collected from the following databases: OMIM (https://www.omim.org/, accessed on 4 June 2025), GeneCards (https://www.genecards.org/, accessed on 4 June 2025), TTD (https://db.idrblab.net/ttd/, accessed on 4 June 2025), DrugBank (https://go.drugbank.com/, accessed on 4 June 2025), ), MalaCards (https://www.malacards.org/, accessed on 4 June 2025), and DisGeNET (https://disgenet.com/, accessed on 4 June 2025). To standardize protein action targets, the UniProt database was used to harmonize the information.

#### 2.1.2. Construction of a Protein-Protein Interaction (PPI) Network for Common Targets Between Juglone and Colorectal Cancer (CRC)

Venn diagrams were used to identify common targets between Juglone and CRC. STRING (https://cn.string-db.org/, accessed on 4 June 2025) was used to obtain protein–protein interaction (PPI) information. The common targets were entered into STRING 12.0, with “Homo sapiens” selected as the species and a confidence level of 0.4 to gather comprehensive protein interaction data. These data files were then downloaded and imported into Cytoscape (Version 3.10.2) software (https://cytoscape.org/, accessed on 4 June 2025) to analyze protein interaction strength. In Cytoscape, the interaction strength of the proteins was calculated using network analysis. This analysis included metrics such as the intermediate degree (BC), compactness (CC), and average shortest path length (ASPL). These metrics indicate the number of connections of a node to other nodes, the role of nodes in the network, their proximity to the network center, and the shortest path length between nodes [15]. This allows for a comprehensive assessment of the network structure. Core targets were screened to identify the active ingredient-target network of Juglone for CRC treatment. As a reference mentioned, we chose the top three targets for molecular docking according to the degree value and screened out the targets with the highest affinity for validation [18].

#### 2.1.3. Gene Ontology (GO) and Kyoto Encyclopedia of Genes and Genomes (KEGG) Enrichment Analyses

The GO database (https://www.geneontology.org/, accessed on 4 June 2025) is used for the identification of drug-disease signaling pathways. The Metascape database (https://metascape.org/gp/index.html, accessed on 4 June 2025) is an effective tool for comprehensive analysis and interpretation. It utilizes the KEGG database (https://www.genome.jp/kegg/, accessed on 4 June 2025) to understand genes and genomes by including the latest gene function annotations, providing systematic gene function analysis [19]. Thus, we imported the Juglone and colorectal cancer intersection genes into the GO database, selected “Homo sapiens” as the species, and ran the program to export the results and associated images. By combining GO and KEGG enrichment analyses, functional information for numerous genes can be obtained from a broad perspective, allowing the identification of drug-disease signaling pathways. The common targets processed by STRING were imported into the Metascape database for GO and KEGG pathway enrichment analyses. This provided data on cellular components (CC), molecular functions (MF), biological processes (BP), and KEGG pathways.

#### 2.1.4. Molecular Docking

To enhance the accuracy of the prediction results, we performed molecular docking of Juglone with the corresponding proteins. The top three proteins, based on their degree value, were selected from the PPI network map. In TCMSP, we entered the naphthoquinone CAS number, downloaded the small molecule MOL2 file, and then searched for “*TP53*” in UniProt. Selected “Homo sapiens” to obtain relevant protein information. We entered this information into the RCSB PDB (https://www.rcsb.org/pages/about-us/index, accessed on 4 June 2025), selected “Homo sapiens”, and then filtered for structures from the last four years with the lowest refinement resolution to select the appropriate protein structure. AutoDock Tools determined the binding site by setting the 3D coordinates of the receptor protein pocket, which improved result accuracy. We then used AutoDock Tools to add polar hydrogens, assign charges, and adjust the grid box. Finally, output the results in the QDBPT format, which is ready for docking. PyMOL is an open-source molecular modeling and visualization software used to analyze and visualize the binding of receptor proteins and ligands. Based on the characteristics of the software and platforms used, we first found the appropriate protein structure, then we performed docking with AutoDock 1. PyMOL 3.0 was used to plot the docking results.

## 3. Experimental Verification

### 3.1. Experimental Material

#### Reagents and Instruments

The CT26 cell line and CT26.WT (cell-specific medium) was purchased from Wuhan Punosai Biotechnology Co., Ltd. (Wuhan, China). Juglone was obtained from Chengdu Phytochemical Pure Biotechnology Co. (Chengdu, China). PBS cell-specific buffer was from White Shark Biologicals (Hefei, China). The antibodies used include p53 Antibody, β-actin Antibody, and HRP goat Anti-Rabbit IgG. Molecular weight standards (10−180 kDa) and SDS-PAGE Tricine Protein Sampling Buffer 2X were sourced from Doctoral Biotechnology Co., Ltd. (Wuhan, China). PAGE Gel Rapid Preparation Kit was obtained from Aase Bio (Shanghai, China). Other reagents included DMSO (Beijing BioTopda Science and Technology Co., Ltd., Beijing, China), Cell Counting Kit-8 (CCK-8) from Beijing Solebo Technology Co., Ltd. (Beijing, China), 0.25% Trypsin-Ethylenediaminetetraacetic Acid (EDTA) Solution from Shanghai Biyuntian Biotechnology Co., Ltd. (Shanghai, China), and ECL Detection Reagent from Boulder Biotech Co., Ltd. (Wuhan, China). The reactive oxygen species detection kit was purchased from Biyuntian Biotechnology Co., Ltd. (Shanghai, China). The apoptosis test kit was purchased from Lian Ke Biotechnology Co., Ltd. (Hangzhou, China).

### 3.2. Cell Cultivation

CT26 cells were maintained in RIMI-1640 medium supplemented with 10% FBS and 1% P/S solution. They were incubated in a humidified chamber at 37 °C with 5% CO_2_. The medium was changed every two days, and cell conditions were monitored. Before each experiment, the cultures were washed with phosphate-buffered saline (PBS), treated with 0.25% trypsin-EDTA solution, and centrifuged at 1000 rpm for 5 min before resuspension.

### 3.3. Cell Viability Assay

CCK-8 assays measure cell proliferation and toxicity by utilizing cellular dehydrogenase to reduce compounds into colored formazan, with absorbance reflecting cell viability. We chose the CCK-8 method to assess the effect of Juglone on cell viability [20]. The advantages of the method are high sensitivity, wide range, high reliability, and high repeatability. CT26 cells were seeded in 96-well plates at a density of 1 × 10^6^ cells/well. After 24 h of incubation, the medium was replaced with varying concentrations of Juglone diluted in culture medium. After 48 h, 10 μL of CCK-8 solution (Biyun Tian, Shanghai, China) was added to each well. Absorbance at 450 nm was measured using an ELISA reader after 2 h of incubation at 37 °C. Each condition was tested in triplicate. Cell viability (%) was calculated and plotted to show the dose-response relationship.

### 3.4. Cell Apoptosis Assay

Apoptosis reagent kits were used to measure cell apoptosis. Cells (2 × 10^5^/well) were seeded into 60 mm culture dishes and treated with Juglone (0, 5, 10, and 20 μmol/L) for 12 h. Approximately 1−1 million cells from each sample were collected in 1.5 mL centrifuge tubes, and the supernatant was discarded after centrifugation. The cell pellet was resuspended in 0.8–1 mL of cell staining buffer. Five microliters of Hoechst staining solution and five microliters of PI staining solution were added, mixed, and incubated in an ice bath or at 4 °C for 20−30 min. Early and late apoptosis in the cells was detected by flow cytometry (BD, New York, NY, USA) [21]. All samples were measured in triplicate.

### 3.5. Detection of ROS

Use the ROS detection kit to measure ROS levels. Seed cells in 60 mm culture dishes (2 × 10^5^/well) and incubate at 37 °C for 24 h. In short, treat the cells with Juglone (0, 5, 10, and 20 μmol/L) for 12 h, then harvest. After washing with PBS, resuspend the cells in DCFH-DA dilution (10 μM, serum-free medium: DCFH-DA = 1000:1) and incubate in the dark at 37 °C for 30 min, then wash three times with serum-free medium. Finally, measure the 2′,7′-dichlorofluorescin (DCF) fluorescence intensity of the cells by flow cytometry (BD, USA).

### 3.6. Western Blot

Cells were divided into four groups: a blank group, a low dose group (5 μmol/L), a medium dose group (10 μmol/L), and a high dose group (20 μmol/L) of Juglone. After 48 h of treatment, CT26 cells were collected, lysed with RIPA lysis solution (Doctoral, Wuhan, China), and proteins were extracted according to the Protein Extraction Kit instructions. Protein concentrations were measured using a BCA assay kit (Thermo Fisher Scientific, Waltham, MA, USA). Proteins were separated by 10% SDS-PAGE and transferred to PVDF membranes (Doctoral Biotechnology Co., Ltd., Wuhan, China). After blocking with 5% (*v*/*v*) blocking solution for 2 h, the membranes were incubated overnight at 4 °C with p53 antibodies (1:1000). Membranes were washed with TBST (Biyun Tian Biotechnology Co., Ltd., Shanghai, China) and incubated with HRP Goat Anti-Rabbit IgG antibody (1:2000) for 2 h at room temperature. ECL luminescence detection was used, and ImageJ software (V1.8.0.112) was employed to analyze the intensity of protein bands and calculate the relative expression of target proteins.

### 3.7. Statistical Analysis

One-way analysis of variance (ANOVA) is used to analyze the relationship between classification data and quantitative data. ANOVA requires that the data satisfy the following two basic conditions: the population of each observed variable should follow a normal distribution, and the population of each observed variable should satisfy the balance of variance. In the actual study, if the absolute value of kurtosis is less than 10 and the absolute value of skewness is less than 3, or the normal graph is basically a bell shape, this indicates that the data are not absolutely normal. All data in this study were statistically analyzed using GraphPad Prism 8.0.0 software (GraphPad Software, San Diego, CA, USA). Data were expressed as mean ± standard deviation and compared using Student’s *t*-test or one-way ANOVA for 2.0 data processing, and multiple comparisons to measure the *p* value of differences between groups. *p* < 0.05 was considered statistically significant.

## 4. Results

### 4.1. Network Pharmacology Analysis

#### 4.1.1. CRC Gene Collection

Targets related to colorectal cancer were identified by searching the OMIM (https://omim.org/, accessed on 4 June 2025), TTD (http://db.idrblab.net/ttd, accessed on 4 June 2025), GeneCards (https://www.genecards.org/, accessed on 4 June 2025), MalaCards (https://www.malacards.org, accessed on 4 June 2025), DIGSEE (http://210.107.182.61/geneSearch, accessed on 4 June 2025), and DisGeNET (https://www.disgenet.org/, accessed on 4 June 2025) databases using “colorectal cancer” as the keyword. The DrugBank database was accessed to identify clinical drug targets for treating colorectal cancer. In the GeneCards database, a higher score indicates a stronger correlation between the target and the disease. Targets with scores above the median in GeneCards and those with scores of 0.1 or higher in the DisGeNET database were considered potential targets for colorectal cancer. The targets for colorectal cancer were identified by merging the information from these databases and removing duplicates. Genes associated with colorectal cancer (CRC) were collected from several databases: 501 from OMIM, 31 from DrugBank, 88 from TTD, 17 from GeneCards, 1206 from MalaCards, 684 from DIGSEE, and 166 from DisGeNET. After analyzing these targets and removing duplicates, a total of 2391 unique genes related to CRC were identified.

#### 4.1.2. Network Diagram of Juglone Targets

**Figure 1:**
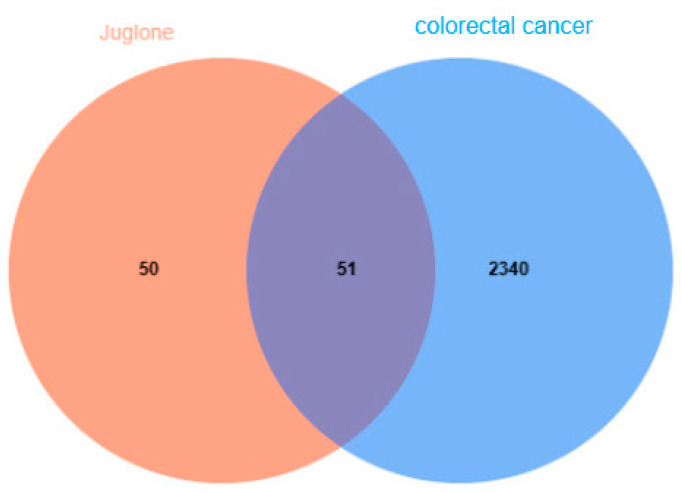
Network diagram of compound targets for active ingredients and potential colorectal cancer targets.

After obtaining the CAS number (481−39−0), name, and Mol ID of Juglone monomers from TCMSP, potential targets for the active ingredients were identified using the SwissTargetPrediction database. These targets were then intersected to determine potential targets and gene information related to Juglone for colorectal cancer treatment. Following data collation, 101 targets were identified, which were compared with disease-related genes. The target information was validated using the UniProt database. Venn diagram analysis of the CRC gene data and potential Juglone targets revealed 51 targets specifically related to Juglone for colorectal cancer treatment (Figure 1). Subsequently, a network diagram illustrating the roles of Juglone targets was constructed using Cytoscape.

#### 4.1.3. PPI Network Construction for Active Ingredients and Disease Common Targets

**Figure 2:**
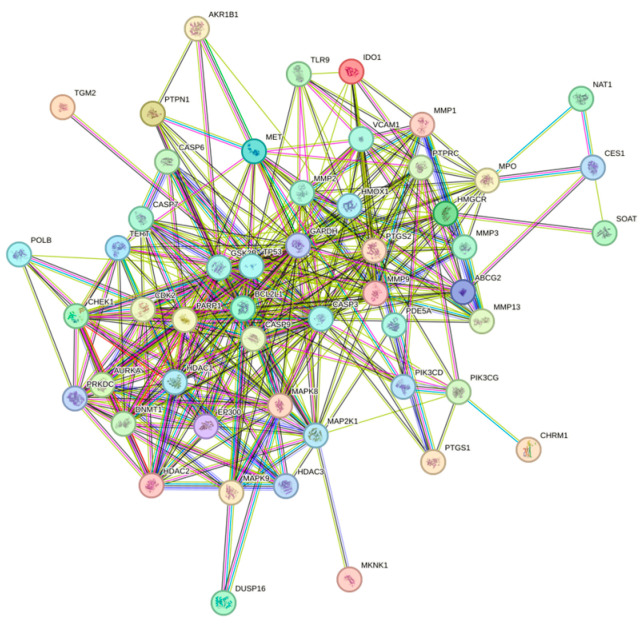
PPI network of genes associated with colorectal cancer treated with Juglone.

**Figure 3:**
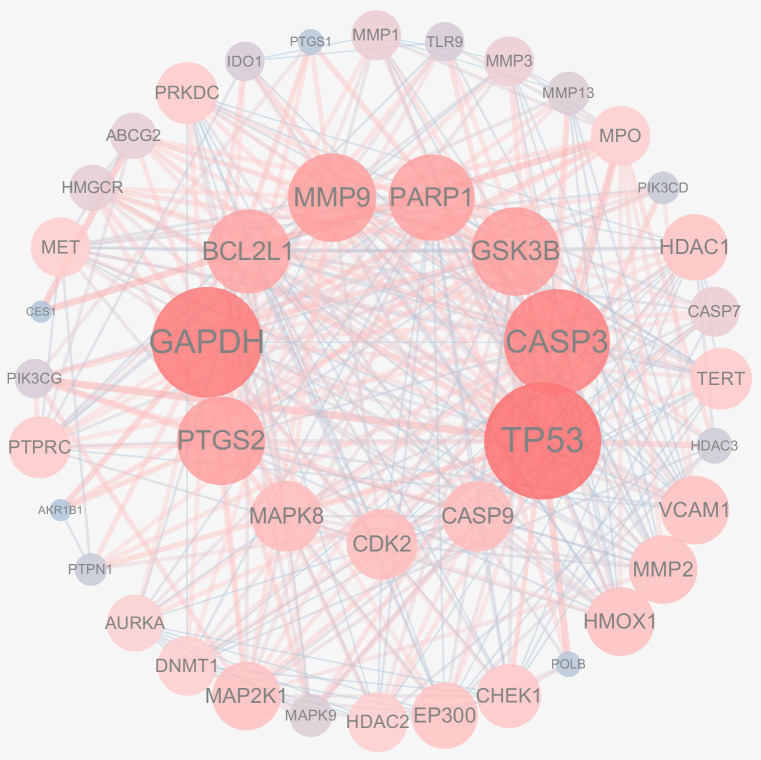
CytoNCA-based results identifying key targets in PPI networks.

The 51 crossover genes were imported into STRING, and a protein–protein interaction (PPI) network for CRC treatment with Juglone was constructed using Cytoscape, as shown in Figure 2. The key targets of the PPI network were identified using the CytoNCA algorithm, which considered Degree, Betweenness Centrality, and Closeness Centrality. The key targets were *TP53*, *CASP3*, *GAPDH*, *MMP9*, *PARP1*, *BCL2*, *BCL1*, *PTGS2*, *GSK3B*, *CDK2*, *CASP9*, and *MAPK8*. Protein nodes were represented by degree, with yellow indicating a higher degree and blue indicating a lower degree. Additional parameters, including connectivity, were used to refine the selection, identifying *TP53*, *CASP3*, and *GAPDH* as targets with higher affinity, as shown in Figure 3. We adjust the size and color of the nodes in the network based on the degree value according to the degree of the nodes. In the graph, darker colors represent greater correlations. We found that the key target with the largest degree value was p53, and we are confident that the association of Juglone with p53 protein.

#### 4.1.4. GO and KEGG Enrichment Analysis

**Figure 4:**
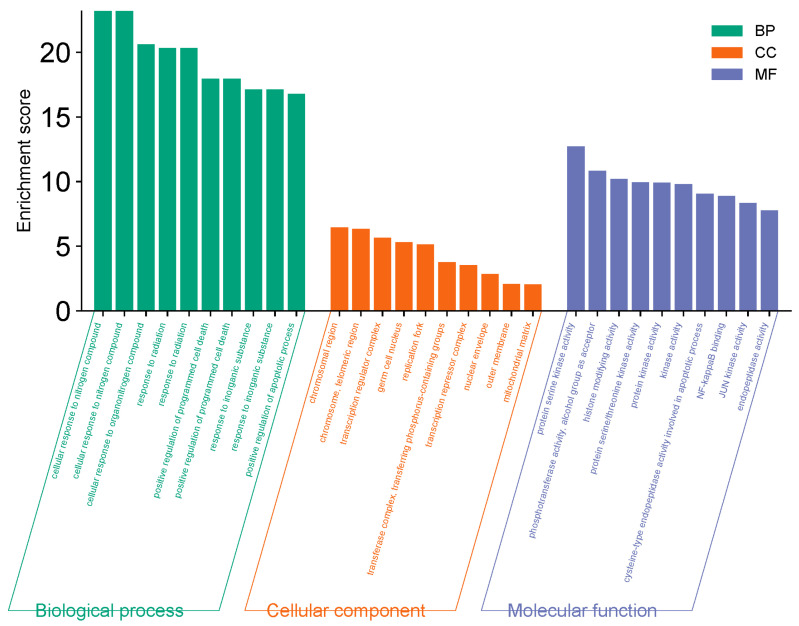
GO function enrichment analysis.

**Figure 5:**
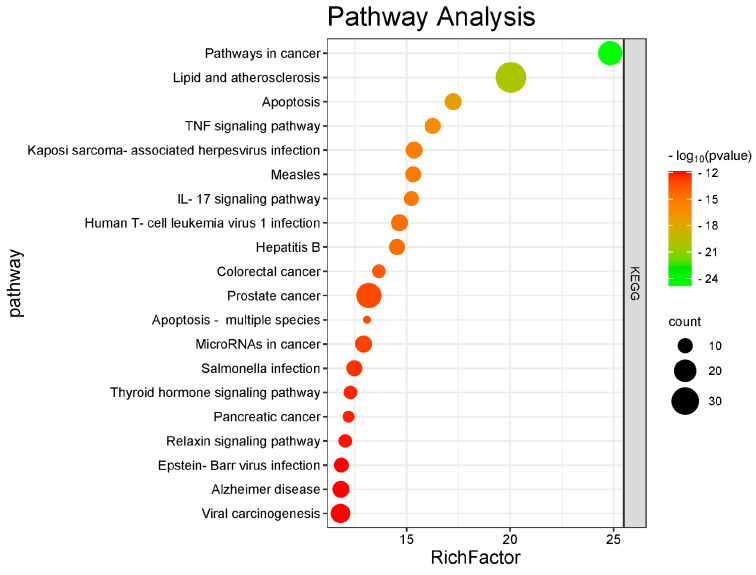
KEGG enrichment analysis (www.kegg.jp/kegg/kegg1.html, accessed on 4 June 2025).

**Figure 6:**
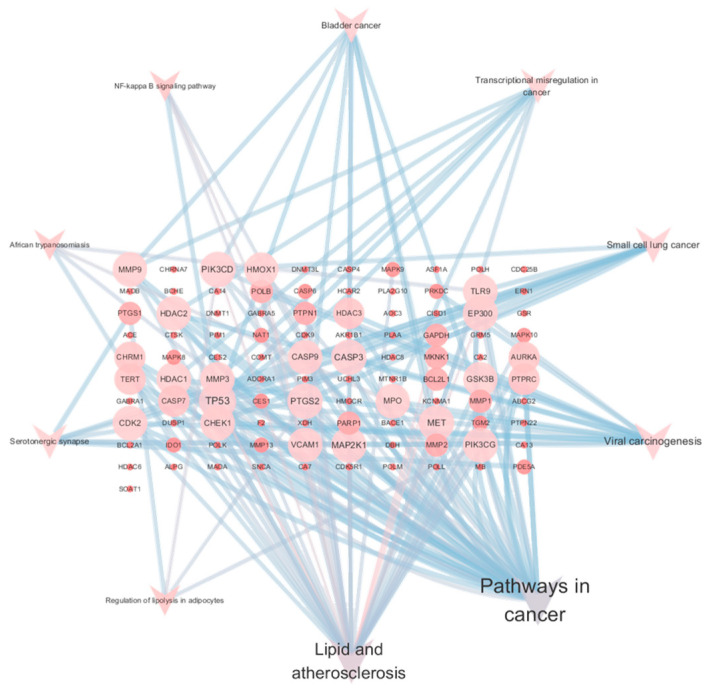
Network map of signaling pathways targeted by Juglone in colorectal cancer.

The 51 predicted gene targets of Juglone for CRC treatment were analyzed for Gene Ontology (GO) enrichment. The analysis revealed 422 biological processes (BP), 23 cellular components (CC), and 77 molecular functions (MF). The top ten processes with the smallest *p*-values (*p* < 0.05) were selected, highlighting key biological activities such as mitochondrial matrix regulation, chromosome organization, immune response activation via cell surface receptor signaling pathways, and phosphohydrolysate hydrolase activity. These results are shown in Figure 4. The KEGG pathway enrichment network diagram was used to visualize the relationships between targets and pathways. The analysis identified 156 pathways, suggesting that Juglone primarily influences CRC development through the regulation of the actin cytoskeleton, Rap1 signaling pathway, axon guidance, protein processing in the endoplasmic reticulum, cGMP-PKG signaling pathway, JAK-STAT signaling pathway, mTOR signaling pathway, phospholipase D signaling pathway, stem cell pluripotency regulation, NK cell-mediated cytotoxicity, and Th17 cell differentiation. The top 10 pathways with significant enrichment (*p* < 0.05) are shown in Table 1 and Figure 5. Cytoscape was used to create a component-target-pathway map, illustrating the associations among these elements based on degree values, as shown in Figure 6. This indicates that Juglone might contribute to CRC treatment through these pathway interactions. Thus, Juglone may exert its therapeutic effects on colorectal cancer by regulating these 51 key genes enriched in the pathways. Juglone may inhibit CRC cell proliferation by targeting specific elements within these cancer pathways.

#### 4.1.5. Analysis of Molecular Docking Results

**Figure 7:**
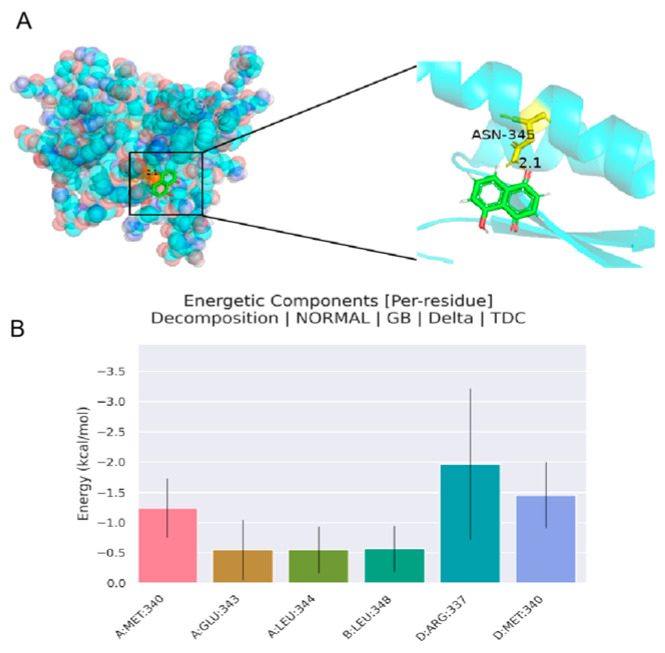
Molecular docking of juglone to the p53 protein and the energy of amino acid residues. (**A**): Juglone docking with p53 protein molecule; (**B**): Energy analysis of relevant amino acid residues.

Based on the PPI network target map, we identified the top three targets with the highest degree values. Molecular docking of Juglone with these targets revealed that Juglone had the strongest binding affinity with the p53 protein. This suggests that increased interaction probability between small-molecule ligands and protein receptors may enhance the regulation of colorectal cancer cell proliferation by inducing structural changes in target proteins. Visualization using PyMol software showed that hydrogen bonds are formed during the binding process, providing specific intermolecular recognition. These findings support the role of P53 as a core target for the treatment of colorectal cancer with Juglone or for mitigating the effects of CRC. Molecular docking results are shown in Table 2 and Figure 7. We found that p53 had the lowest binding energy, kcal/mol, which indicated that p53 was a key protein for the anti-tumor effect of Juglone, and the foundation to explore the mechanism of Juglone to inhibit the value added of colorectal cancer cells.

#### 4.1.6. Analysis of Molecular Dynamics Simulation Results

**Figure 8:**
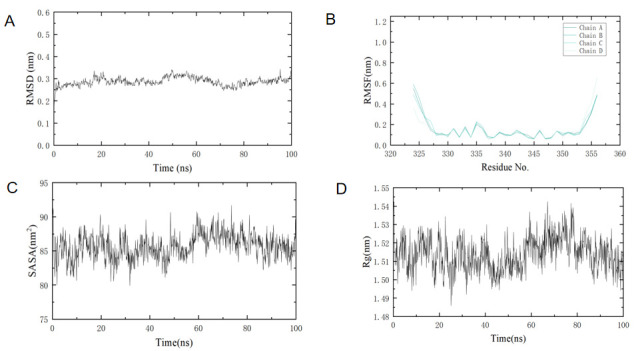

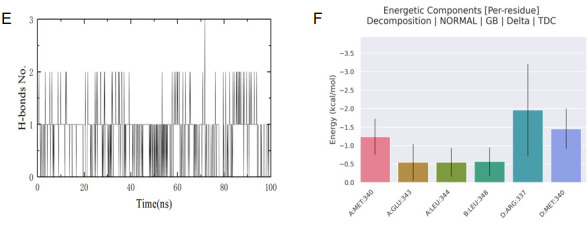
Important parameters of proteins and ligands in molecular dynamics simulations. (**A**) RMSD values for the p53 protein complex; (**B**) RMSF values for the p53 protein complex; (**C**) SASA values for the three complexes; (**D**) Rg values for the p53 protein complexes; (**E**) the number of hydrogen bonds in the p53 protein complexes; (**F**) contribution of a single amino acid residue to binding free energy.

After about 10 ns of molecular dynamics simulations of the three protein complexes, as shown in Figure 8, the RMSD value of the system as a whole stabilized at about 0.3 nm, indicating that the molecules of the complex have reached a stable state. Based on the RMSF value, the flexibility of the protein residue can be judged, and the residue part with a higher RMSF value is also more flexible, and the Rg (radius of gyro) and SASA (solvent accessible surface area) values tend to be stable, indicating that the structure of the protein and ligand complex is very stable. In addition, all three protein complexes have a lower free energy state, representing a more stable state that the system tends to achieve.

### 4.2. Analysis of Cell Cytotoxicity and Proliferation Assay Results

#### 4.2.1. The Anti-Proliferative and Pro-Apoptotic Effects of Juglone on CT26 Cells

**Figure 9:**
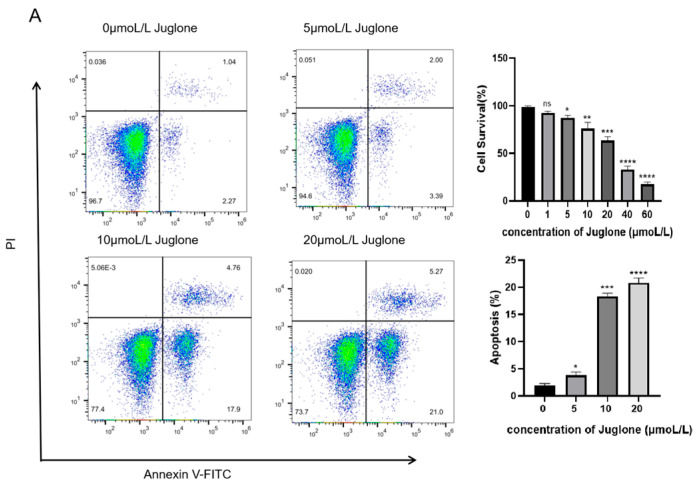

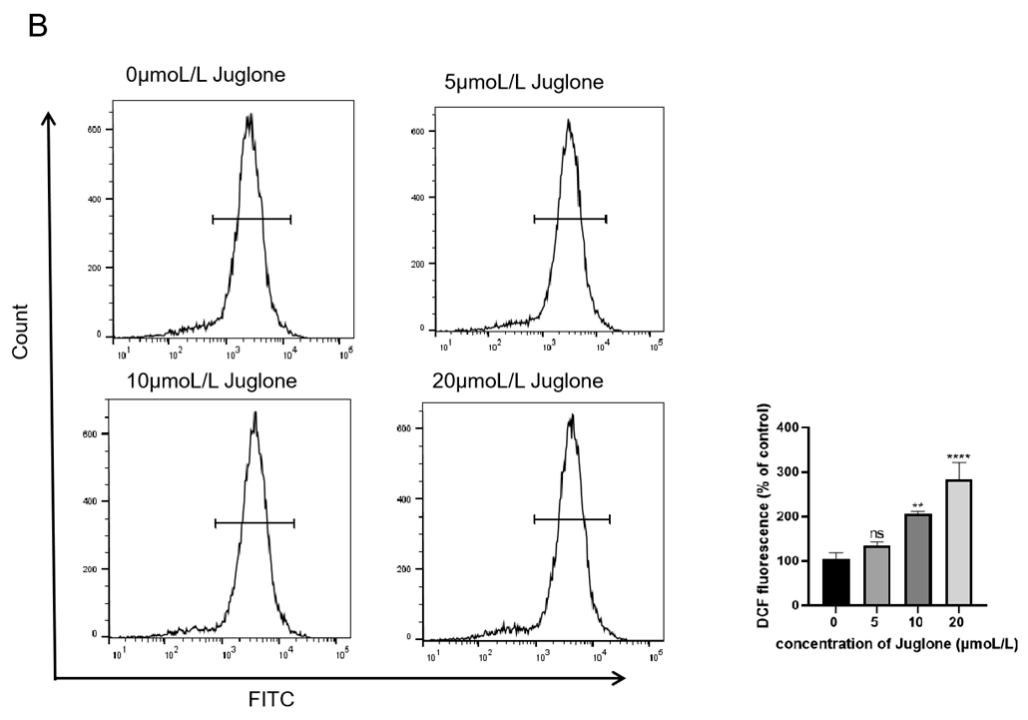
Flow cytometry was used to detect the apoptosis and ROS of CT26 cells treated with ubiquinone for 12 h. (**A**) CT26 was treated with Juglone (0, 5, 10, 20 µmol/L) for another 12 h. Apoptosis was detected by flow cytometry; Effect of different concentrations of carnosine on CT26 colorectal cancer cells. (**B**) CT26 cells were treated with different Juglone concentrations for 12 h, and ROS levels were detected by flow cytometry. Data are presented as mean ± SD (*n* = 3). * *p* < 0.05, ** *p* < 0.01, *** *p* < 0.001 and **** *p* < 0.0001 indicate significant differences between the Juglone (0 μmol/L) groups, ns stands for *p* > 0.05.

To determine the effect of Juglone on colorectal cancer cells, we measured the viability of CT26 cells treated with various concentrations of Juglone using the CCK—8 assay. Data analysis with GraphPad Prism software (Version 8.0) revealed that the 20 μmol/L group of Juglone significantly inhibited cell viability compared to the other groups (** *p* < 0.01). The cell viability of the Juglone group gradually decreased with the increase of Juglone concentration. When the Juglone concentration was 40 μmol/L, the survival rate of CT26 cells was only 30.71%, so we selected 5 μmol/L, 10 μmol/L, and 20 μmol/L for follow-up studies(Figure 9A). We detected Juglone-induced apoptosis using flow cytometry to determine whether the anti-proliferative effect of Juglone on CT26 cells is related to apoptosis. As shown in Figure 9A, after 12 h of intervention, Juglone induced apoptosis in CT26 cells in a dose-dependent manner. The apoptosis rate of CT26 cells treated with 10 μmol/L of Juglone increased. The results indicate that Juglone can indeed induce apoptosis in CT26 cells.

#### 4.2.2. ROS Production Induces Cell Apoptosis in CT26 Cells

DCFH-DA fluorescent probe was used to measure the effect of different concentrations of Juglone on ROS generation. As shown in Figure 9B, Juglone increases the fluorescence intensity in a dose-dependent manner, indicating that Juglone induces the accumulation of ROS during the treatment of CT26 cells.

#### 4.2.3. CT26 Cells Increase p53 Protein Expression to Promote Cell Apoptosis

**Figure 10:**
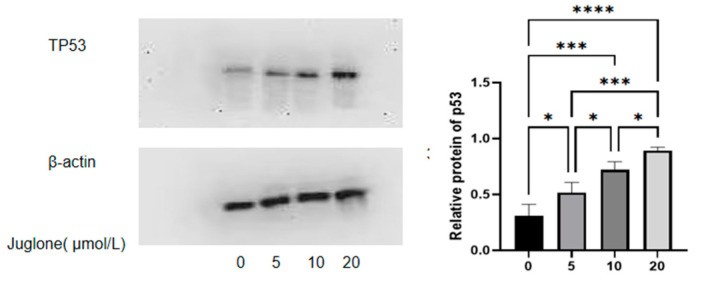
Expression of p53 protein. Compared with 0 μmol/L, * *p* < 0.05, *** *p* < 0.001, **** *p* < 0.0001.

Western blot analysis was used to detect the expression of p53 protein in CT26 cells treated with various concentrations of Juglone, as shown in Figure 10. The cells of the control group, which were not treated with any drug or with Juglone, were labeled as 0 μmol/L in the first lane of the Western blot results. The results indicated that treatment of the CRC cell line with 5 μmol/L, 10 μmol/L, and 20 μmol/L Juglone had significantly increased the expression of p53 protein in a dose-dependent manner compared to untreated (0 μmol/L) cells. This suggests that Juglone can inhibit tumor cell growth by modulating protein expression in CT26 cells.

## 5. Discussion

Colorectal cancer is now one of the most devastating malignancies globally. As of 2020, both the incidence and mortality rates of colorectal cancer are rising in China [22]. This situation is severe, highlighting an urgent need for a low-toxicity, effective drug for clinical use. Juglone, a compound from the naphthoquinone group, is extracted from the walnut plant. It is sourced from the roots, bark, leaves, and green shells of the walnut tree. Current research indicates that Juglone affects the proliferation, growth, and apoptosis of tumor cells [23,24]. In vitro experiments have demonstrated that Juglone inhibits both the proliferation and colony formation of Caco-2 colorectal cancer cells. Animal studies have shown that Juglone reduces tumor volume in tumor-bearing mice [25]. Researchers have shown through in vitro studies that Juglone derivatives exhibit dual inhibitory effects on both tumor cells and tumor cell-induced platelet proliferation [26]. However, there are no relevant reports on the research mechanism of Juglone against colorectal cancer.

In the process of drug development, information biology and computer science have shortened the exploration time and cost related to experimental targets and mechanisms in drug development [27]. In this experiment, we applied network pharmacology to screen out 51 targets in GO analysis, and we found that Juglone is closely related to the tumor pathway, suggesting that Juglone can have a therapeutic effect on colorectal cancer through this pathway in order to explore the specific effect correlation of Juglone, we selected the p53 protein with the strongest association according to the degree value were docked to small molecules (Juglone), and the binding energies of these three proteins to small molecules remained low in the free energy state, and the whole complex system tended to be stable. To further analyze the complex interactions and their stability, we performed dynamic simulations of the p53 protein complexes. By calculating the RMSD and RMSF of the protein–ligand complex, after approximately 10 ns of MD simulation, The RMSD value of the system is stable at about 0.3nm, indicating that the molecule of the complex has reached a stable state, the residue part with a relatively high RMSF value has a great degree of flexibility, and it is normal for the amino acid structure of the protein to fluctuate greatly at the beginning and tail ends of the protein, and on the whole, the structure of the protein and ligand complex was very stable [28]; Rg can indicate the overall tightness of the protein during the simulation process, SASA describes the surface area of the protein exposed to solvents during the simulation process; these two parameters are very important for evaluating the stability of the protein [29]. Through network pharmacology, our molecular docking results found that the p53 protein is closely related to colorectal cancer, while the molecular dynamics simulations evaluated the parameter values of RMSD, RMSF, Rg, and SASA, further confirming the stability of the p53 protein and juglone complex. Therefore, we believe that the ability of Juglone to combat colorectal cancer is related to the regulation of p53 protein expression.

p53 regulates cell division, prevents the proliferation of mutant or damaged cells, and transmits apoptosis signals through transcriptional regulation, thus inhibiting tumor formation. A clinical study assessed how p53 protein expression levels affect the efficacy of adjuvant chemotherapy in stage III colorectal cancer patients. The results indicated that adjuvant chemotherapy was more effective in patients with higher p53 protein expression levels [30]. The p53 protein can inactivate cells by regulating related genes, cycle arrest, and other ways to cause apoptosis of tumor cells. It has been demonstrated that p53 can exert anti-tumor effects by modulating myeloid leukemia factor 2 (MLF2) [31]. In one study, it was found that by enhancing the expression of Tripartite motif (TRIM) family proteins, p53 activity increased, making colorectal cancer cells sensitive to chemotherapy and indirectly accelerating tumor cell apoptosis [32]. Many tumors carry mutations in the *TP53* gene, leading to the loss of function of the encoded protein. *TP53* is one of the most commonly mutated genes in all cancers, with *TP53* mutants occurring in more than 40% of colorectal cancer (CRC). Accumulation of mutant p53 may enhance colorectal cancer stem cell (CCSC) phenotype and enhance colorectal tumorigenesis [33]. Therefore, lowering the levels of mutant p53 protein is an effective anti-cancer strategy. ROS is a key factor in the occurrence and development of tumors. Its excessive accumulation in cells induces apoptosis. The extract from bitter tea can increase the accumulation of ROS, inducing apoptosis in tumor cells [34]. The p53 protein plays a key role as a negative regulator in the glycolysis of tumor cells, thereby reducing the levels of reactive oxygen species (ROS) within the cell, which helps the programmed cell death of cancer cells [35,36]. ROS also mediates crosstalk between extrinsic and intrinsic pathways, triggering p53-dependent apoptosis in HCEP (Human Corneal Epithelial Cell) cells [37]. Colorectal cancer is a multifactorial disease related to age, and the disruption of the p53 pathway can induce early-stage colorectal cancer [38]. Wu et al. found that the proteoglycans extracted from Ganoderma lucidum can regulate ROS levels and the autophagy process to promote apoptosis in human pancreatic cancer cells [39]. Here, the expression level of p53 protein in CT26 cells treated with Juglone increased, the ROS level significantly rose, and the apoptosis rate of CT26 cells increased. These results indicate that Juglone can elevate ROS levels to induce apoptosis, and the key target for apoptosis is related to the expression level of THE p53 protein.

## 6. Conclusions

Experimental studies indicate that Juglone’s therapeutic effects on colorectal cancer are associated with the top 10 signaling pathways and 51 potential target genes. p53 exhibits the highest protein–protein interaction frequency within the complex gene network. Molecular docking further confirmed the strong affinity between TP53 and active components. Cell-based experiments demonstrate that Juglone promotes apoptosis in CT26 cells by increasing the accumulation of ROS, with key apoptotic targets being related to the expression of the p53 protein. There is growing evidence that Juglone is a potent anti-cancer molecule of plant origin. However, its anti-cancer effects on colorectal cancer cells have not been fully explored. In this experiment, we explored the anti-colorectal cancer mechanism of Juglone through computer science and cellular experiments, providing a reference for the research on anti-tumor drugs while also laying the foundation for the development and utilization of Juglone. However, in this study, the mechanism of action of Juglone against colorectal cancer was only verified in vitro, and there were limitations. Therefore, we will continue to investigate the mechanism of action of Juglone in the CRC tumor mouse model to lay the foundation for the screening of anti-cancer drugs.

## Figures and Tables

**Table 1:** Key parameters of network nodes in colorectal cancer targets.

Gene Name	Betweenness Centrality	Closeness Centrality	Neighborhood Connectivity	Radiality	Degree
*TP53*	0.17660225	0.85964912	16.14634146	0.95918367	41
*GAPDH*	0.09942267	0.80327869	16.94736842	0.93877551	38
*CASP3*	0.07554407	0.77777778	17.58333333	0.92857143	36
*GSK3B*	0.04488729	0.68055556	19.03571429	0.88265306	28
*MMP9*	0.03629503	0.69014085	18.85714286	0.8877551	28
*PTGS2*	0.05012007	0.7	18.67857143	0.89285714	28
*PARP1*	0.02609519	0.66216216	19.59259259	0.87244898	27
*BCL2L1*	0.02295318	0.65333333	20.11538462	0.86734694	26
*MAPK8*	0.03274029	0.59756098	21.3	0.83163265	20
*CDK2*	0.00417775	0.59036145	22.6	0.82653061	20

**Table 2:** Binding energy of the main ligand group to the receptor.

Target	Protein Affinity kcal/mol Compound
p53	−5.39
Caspase-3	−4.67
GAPDH	−5.15

## Data Availability

All relevant data are contained within the article. The original contributions presented in the study are included in the article. Further inquiries can be directed to the corresponding author/s in the main manuscript.
